# Dreams are more “predictable” than you think

**DOI:** 10.3389/frsle.2025.1625185

**Published:** 2025-07-23

**Authors:** Lorenzo Bertolini, Sergio Consoli, Julie Weeds

**Affiliations:** ^1^European Commission, Joint Research Centre (JRC), Ispra, Italy; ^2^Department of Informatics, University of Sussex, Brighton, United Kingdom

**Keywords:** dream report analysis, dream reports modeling, gender difference, dreaming in blind participants, machine learning, large language models, natural language processing

## Abstract

**Introduction:**

A growing body of work has used machine learning and AI tools to analyse dream reports, and compare them to other textual content. Since these tools are usually trained on text from the web, researchers have speculated they might not be suited to model dreams reports, often labeled as “unusual” and “bizarre” content.

**Methods:**

We used a set of large language models (LLMs) to encode dream reports from DreamBank and Wikipedia. To estimate the ability of LLMs to model and predict textual reports we adopted perplexity, a measure based on entropy, formally, the exponentiated log-likelihood of a sequence. Intuitively, perplexity indicates how “surprising” a sequence of words is to a model.

**Results:**

In most models, perplexity scores for dream reports were significantly lower than those for Wikipedia articles. Moreover, we found that perplexity scores were significantly different in reports produced by male vs female participants, and between blind and normally sighted individuals. In one case, we found this difference to be significant between clinical and healthy subjects.

**Discussion:**

Dream reports were found to be generally easier to model and predict than Wikipedia articles. LLMs were also found to implicitly encode group differences previously observed in the literature based on gender, visual impairment, and clinical population.

## 1 Introduction

Dream reports describe the content of the conscious experiences we had while asleep. Through the years, researchers have used these transcripts to connect dreams with awakened states (Blagrove et al., 2004; Skancke et al., 2014; Andrews and Hanna, 2020), and to study consciousness (Nir and Tononi, 2010; Siclari et al., 2017) and pathological conditions (Kobayashi et al., 2008; Skancke et al., 2014; Thompson et al., 2015; Andrews and Hanna, 2020). For these reasons, both researchers and practitioners have been consistently interested in dream reports, and have developed a variety of frameworks to study, analyse, and annotate their content in a systematic way (Hall and Van De Castle, 1966; Hauri, 1975; Schredl, 2010).

The analysis and annotation processes of dream reports can be extremely time-consuming and rely upon human experts who usually undergo long training, which has limited the growth and reproducibility of research around dreams and dream reports (Elce et al., 2021). As a result, researchers have shown a growing interest in adopting automatic analysis of dream reports' content and structure, based on machine learning and natural language processing (NLP) (see Elce et al., 2021 for a review). Many of these approaches use models that have been fully, or partially, trained on large amounts of rather standardized text from the internet, such as Wikipedia (Nadeau et al., 2006; Razavi et al., 2013; Altszyler et al., 2017; Sanz et al., 2018; McNamara et al., 2019; Bertolini et al., 2024b,a; Cortal, 2024).

Since a vast body of work identifies dream reports as being more bizarre than wakeful experience (Rosen, 2018), one might assume that training a model on more structured and formal textual data might limit the ability of the said model to deal with reports from dreams—a position informally held by multiple researchers in the community. While the extent to which dream reports quantitatively differ from other forms of textual transcripts remains a matter of significant debate (Kahan and LaBerge, 2011; Domhoff, 2017; Zheng and Schweickert, 2023), multiple studies have indeed shown that their semantic content and word use can significantly diverge from other forms of textual items. Many of these studies are based on dictionary-based frequency analysis of content words (e.g., Bulkeley and Graves, 2018; Mallett et al., 2021; Zheng and Schweickert, 2021; Yu, 2022; Zheng and Schweickert, 2023; Zheng et al., 2024). While fully transparent and computationally efficient, dictionary-based approaches such as LIWC (Pennebaker et al., 2015) do present some critical issues (Bulkeley and Graves, 2018; Zheng and Schweickert, 2023; Bertolini et al., 2024a), such as typographical errors, or limited access to a broader context and syntactic structure. However, multiple works have shown how these methods could be used to discover differences between different types of dreams, such as nightmares, lucid dreams, and baseline dream reports (Bulkeley and Graves, 2018; Zheng and Schweickert, 2023). A partial solution was proposed by Zheng and Schweickert (2023), which expanded on the previous literature by studying the differences between dream reports and other types of textual transcripts, using both LIWC and support vector machines (SVM) (Cortes and Vapnik, 1995). The LIWC approach found a large set of categories that significantly differ between dream and non-dream reports, and the proposed SVM approach could successfully discriminate between the two categories of reports. However, the adopted dataset was quite limited in magnitude—around 800 instances, balanced between dream and non-dream reports. This constraints the generalisability of the findings, largely grounding the observed difference to the dataset of choice. Altszyler et al. (2017) introduced an approach more rooted in the overall semantic content of the textual report, by comparing two word-embedding approaches (namely Latent Semantic Analysis (LSA) (Landauer and Dumais, 1997) and word2vec's skip-gram with negative samples (Mikolov et al., 2013) to investigate how the relationship between a content word like *run* changes in large web corpora compared to a large collection of dream reports from DreamBank (Domhoff and Schneider, 2008). In this work the authors discovered that LSA better encodes the difference in the type of contexts such words appearing in the two types of corpora.

Since sleep and dream research are witnessing an increasing amount of NLP-based approaches, investigating whether these qualitative differences might have a quantifiable impact on NLP models is of crucial importance, as it might limit the ability of such tools to model dream reports, particularly if these methodologies utilize unsupervised techniques. This work proposes to address this specific issue directly. Unlike previous work, which focused on qualitatively identifying *what content* makes a (limited set of) dream and waking reports different (Zheng and Schweickert, 2023; Zheng et al., 2024), we study in a quantitative manner *how much* a (large) set of dream reports appears to be “surprising” to a model that has seen a huge amount of non-dream-based text. To do so, we adopt a fully unsupervised solution based on pre-trained autoregressive large language models (LLMs), and on perplexity, a popular NLP metric, intuitively indicating how well an LLM can predict a sequence of words. The proposed approach has found similar application in Colla et al. (2022) work, where authors showed how perplexity scores from GPT2 (Radford et al., 2019) and n-*grams* can be used to discriminate between healthy participants and patients with Alzheimer's disease.

This work makes four main contributions. First, it shows that, when considered as a continuous string of text, (a large proportion of) DreamBank is only marginally harder to predict than (a comparable section of) Wikipedia. Second, and most importantly, dream reports are on average significantly more predictable than Wikipedia articles when considered as single textual units. Third, it identifies a negative correlation between the number of words in a report/article and how “surprising” such a report/article appears to the model. Fourth, it provides preliminary evidence suggesting that gender and visual impairment can significantly impact how “surprising” a report appears to the model, providing the first evidence that modern NLP tools such as LLMs internally and implicitly replicate group differences previously observed in the literature.

## 2 Materials and methods

### 2.1 Metric and models

The primary interest of this work is to quantitatively assess whether dream reports are in fact harder to model and predict for a pre-trained large language model (LLM), the current tool of choice in most NLP research and applications. To measure this phenomenon, we adopt perplexity (PPL) (Huyen, 2019). Intuitively, perplexity can be seen as a measures of how “unpredictable” or “surprising” a given string of text is for a model. In other words, given a target word *i*, and a sequence of words (*c*, for context) preceding *i*, perplexity measures the ability of an LLM to predict *i*, given its context *c*. Lower the perplexity scores, higher is the ability of a model to predicting how a sentence evolves. In other words, low perplexity indicates low surprisal. Formally speaking, perplexity is the exponentiated log-likelihood of a sequence *X* and is computed using Equation 1:


(1) $$\begin{array}{l} PPL\left(X\right)=epx\left\{-\frac{1}{t}\overset{t}{\underset{i}{\sum }}logp_{\theta }\left(x_{i}|x_{<i}\right)\right\}, \end{array}$$

where *X* = (*x*_0_, *x*_1_, ..., *x*_*t*_) is the sequence of words, *logp*_θ_(*x*_*i*_|*x*_ < *i*_) is the log-likelihood, of the *i*^*th*^ word conditioned by the preceding context (*x*_ < *i*_). While many other solutions have been proposed to evaluate how well a language model can capture different linguistic phenomena, perplexity is still widely used and can inform us on how well a model reflects natural language by measuring how distant a string is to a more “natural” sequence (Meister and Cotterell, 2021). Hence, our goal can be stated as understanding whether a machine trained on a very large amount of textual data “perceives” dream reports as “surprising” (i.e., as having a high perplexity).

**Figure 1:**
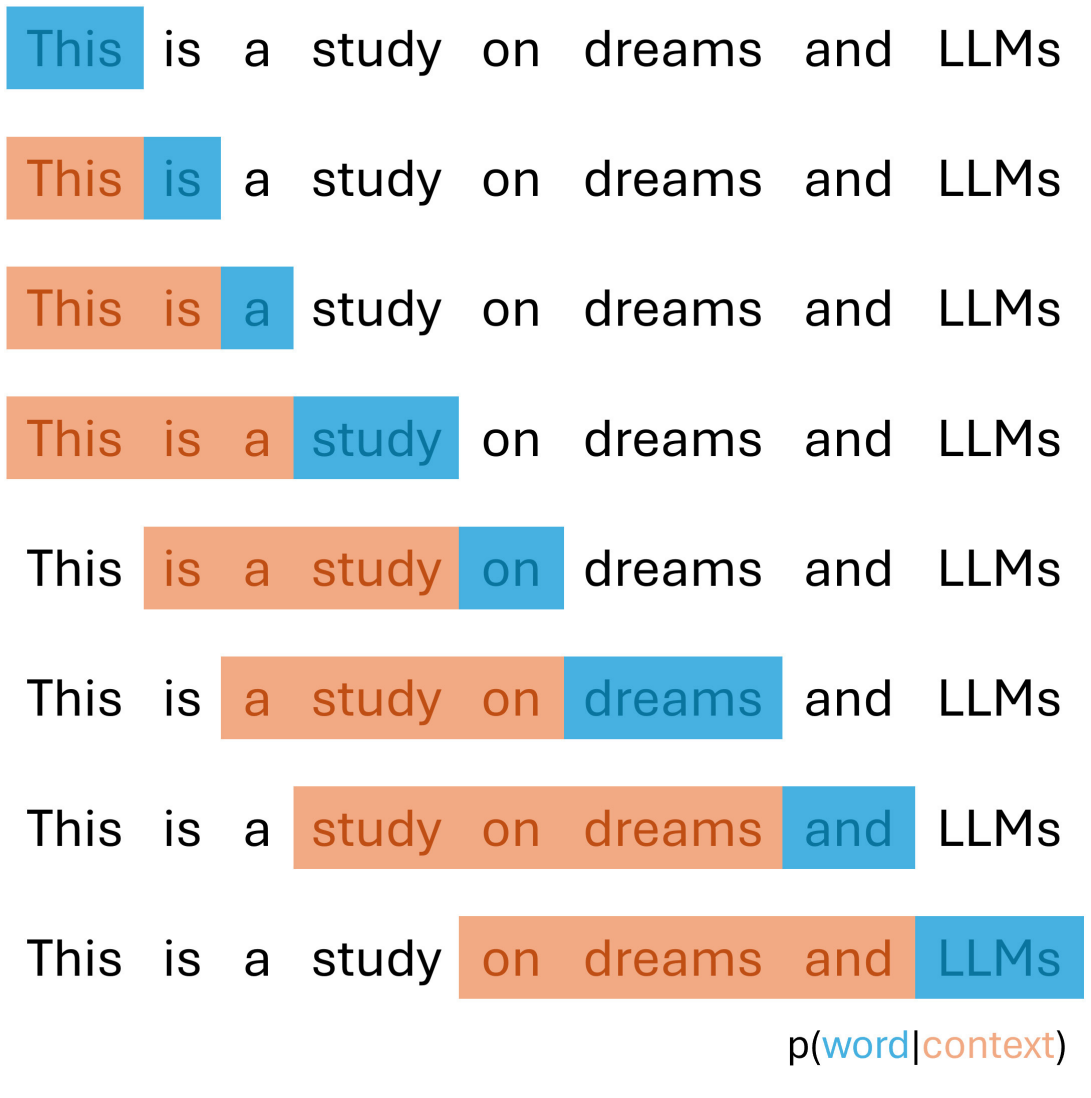
Schematic representation of perplexity computation, using a sliding fixed window context of three words.

For models without computational constraints, perplexity should be evaluated using a sliding-window approach. This method slides the context window across the text, ensuring the model has sufficient context for each prediction. The process sums negative log-likelihoods for all word-context pairs and averages across total words, as shown in Figure 1.

This approach better approximates true sequence probability decomposition and typically produces more favorable scores. However, it requires a separate forward pass for each token, making it computationally expensive. A practical solution uses strided sliding windows, moving the context by larger steps rather than single tokens. This maintains a large context while significantly reducing computation time. Following Hugging Face implementation^1^, we use a stride of 512 tokens with each model's maximum sequence length as input size (context plus target word). These settings surpass the results reported in the original GPT-2 papers.

This strided approach efficiently computes perplexity for large datasets that cannot fit entirely in model memory. For shorter sequences that fit within the model limits, we can process them entirely at once, obtaining a single perplexity score per sequence. Our work primarily uses this single-sequence approach, focusing on individual dream reports and Wikipedia articles. As detailed below, these texts never exceed the maximum input length for any model investigated.

To model our textual data, we adopt models from two series of autoregressive pre-trained LLMs: GPT2 and OLM0 (Groeneveld et al., 2024). The GPT2 family consists of GPT2 (137 million (M) parameters), GPT2-Medium (380 M), GPT2-Large (812 M), and GPT2-XL (1,610 M). On the other hand, the OLMo family presents two models: OLMo-1B (1,180 M), and OLMo-7B (6,890 M).

Indeed, the current landscape of autoregressive LLMs offers a suite of impressive alternatives, such as GPT-4 (OpenAI et al., 2024), Gemini (Team, 2024), or Llama 3 (Dubey et al., 2024). However, our selection of models allows us to control for multiple interesting factors, namely the impact of model size, training data, and the evolving state of the art. While it might seem an era ago—and certainly was in AI terms...—GPT2 once was (at) the pinnacle of the LLM leader-board. Indeed, OLMo's performance is not *extremely* representative of the state of the art. However, at its release time, it was on par with the highest-end competitors, such as Llama 2. Aside from their performance, these two families share an important factor, which makes them more suitable for our experiments than more recent and powerful models: the extent to which we know their training data. Contrary to its more recent siblings, we have quite some information on the data used to train GPT2. Most importantly, on what was *not* used for its training, namely, Wikipedia (Radford et al., 2019). On the other hand, and even more unusual for the current standard, OLMo's training data is *fully* open source. Not only do we know Wikipedia was used for its training, but we can search *which* articles were used in the model training. While documents from Wikipedia compose a little over .1% of the overall documents in training set (Soldaini et al., 2024), this is extremely relevant to our experiments as it allows us to frame the results of the models with respect to their pre-training procedure. Lastly, both families, which have a convenient point of contact in the two one-billion-parameters models, present a set of models growing in size, which nicely reflects the capabilities and approach of the time frame they were built in, and can allow us to study how increasing the number of parameters in a model impacts its ability to model dream and other textual data, depending on its training data. In summary, if the hypothesis that dream reports are harder to model for LLMs, we should find that average PPL scores for dream reports should be, on average, significantly higher than PPL scores for Wikipedia articles, especially in LLMs exposed to Wikipedia's articles during training.

### 2.2 Dataset

#### 2.2.1 Dream dataset

Similarly to previous work (Fogli et al., 2020; Gutman Music et al., 2022; Bertolini et al., 2024a,b; Cortal, 2024), we adopt a set of dream reports extracted from DreamBank (Domhoff and Schneider, 2008)^2^, an online collection of dream reports from different people and scientific studies. The original dataset contains approximately 22k reports in the English language, annotated with respect to gender, year of collection, and series—the specific subsets of DreamBank representing (groups of) individuals from which dreams are collected.

#### 2.2.2 Text dataset

We use Wikipedia as the source of our baseline text. More specifically, we consider the WikiText2 dataset (Merity et al., 2017)^3^, an open-source dataset containing approximately 20k articles from Wikipedia. This specific baseline choice is motivated by two main reasons. First, and more specifically to our models of choice, (part of) Wikipedia is included of OLMo's training set. Moreover, WikiText2 was entirely excluded from GPT2 pre-training, and was instead used as one of the testing benchmarks in the original paper. Second, and on a general stance, adopting Wikipedia allows for a strict comparison with a standardized text, in terms of syntactic and semantic structure. This is due to the fact that large portions of Wikipedia are formally and heavily curated, and can hence work as a “stress” test for the hypothesis that dream reports are notably different.

#### 2.2.3 Sampling

**Figure 2:**
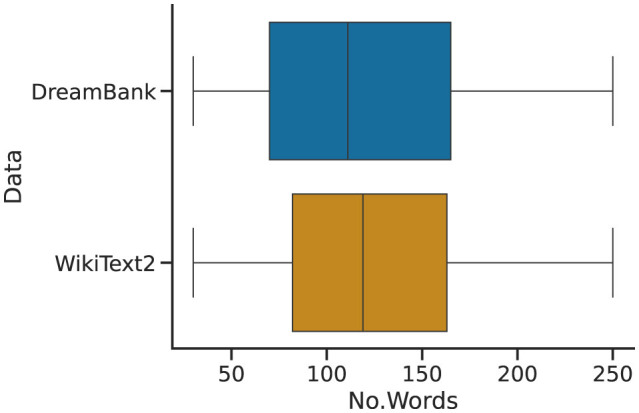
Word-count distributions of the number of words (No. Words) per instance in the final Wikipedia (WikiText2) and dream (DreamBank) test sets used in the experiments.

Given the discrepancy between the datasets' magnitude and some of their specific content, we use a filtering and sampling procedure over the original datasets. We begin by filtering out from Wikipedia all those instances that do not contain an article's body—that is, instances consisting of only titles or empty strings. To limit the possibility that a variable such as the number of words might impact our experiments, we further extract from both DreamBank and Wikipedia the set of items laying that contain between 30 and 250 words. The remaining datasets consist of approximately 13 k Wikipedia articles and 17 k dream reports. To generate a test set with a similar distribution in the number of words per instance, we interactively sample a subset of dream reports of the same magnitude as the remaining Wikipedia set (i.e., 13 k) for 250 iterations. We then run a random permutation test comparing the Wikipedia set against each sample dream set and select the least diverging one. The final distributions are described in Figure 2, and are made freely available (see link in the “data availability statement” section).

### 2.3 Statistical analyses

We compare one-dimensional distributions (e.g., how many words constitute each dream report) with a random permutation test. To assess whether two-dimensional distributions (e.g., the number of words *and* perplexity scores of each Wikipedia article) are significantly different from one another, we adopt the Peacock test, which is a two-dimensional non-parametric generalization of the Kolmogorov-Smirnov test (Peacock, 1983; Fasano and Franceschini, 1987). Correlation analyses are based on Spearman's coefficient. All *p* values in the work refer to scores obtained after applying Holm correction (Holm, 1979), a method used to adjust p-values for multiple comparisons to minimize Type I errors, by sequentially adjusting the significance threshold as a function of the number of tests performed. Experiments were run with the support of an NVIDIA H100 80GB HBM3 GPU. The code and data to replicate the experiments are freely available at https://github.com/jrcf7/report_perplexity.

## 3 Results

### 3.1 Comparing dream reports and Wikipedia articles

Table 1 gives an overview of the overall perplexities produced by the different versions of GPT2 and OLMo on the two test sets, namely DreamBank and WikiText2. The table further contains the respective lengths of the datasets, in terms of the total number of tokens, and the size of each model (in millions of learnable parameters). Based on the results in the table, we can make three main observations. First, the perplexity scores for WikiText2 from our experiments closely resemble those of the original paper that introduced the GPT2 models (Radford et al., 2019). Second, compared to DreamBank, each model seems to produce lower perplexity scores for Wikipedia. Third, while the perplexity scores produced by GPT2 on WikiText2 and DreamBank appear close to each other (31.9 vs 27.4), the distance grows with model size. Although in a smaller magnitude, a similar trend is observed for the two variants of OLMo. Interestingly, in these models, the differences between datasets are not as marked as they are for GPT2 models. This behavior is unexpected since (part of) Wikipedia is included in OLMo's training set, and should hence have a significant advantage over out-of-distribution data like dream reports. This evidence could suggest that WikiText2, or part of it, might not be part of the Wikipedia subset used to train OLMo models. Overall, the differences remain relatively small across the board of the GPT2 models. Moreover, whilst the perplexity of DreamBank is overall higher than that of WikiText2, this discrepancy might be explained by the fact that DreamBank is built from collections of very different individuals, from (very) different time periods. In other words, while Wikipedia articles tend to follow a more unified language type and structure, DreamBank's reports can suddenly and significantly vary from one line to the other.

**Table 1:** Whole corpora results.

**Data**	**Model**	**Perplexity**	**Dataset length (M)**	**Model size (M)**	**Original PPL**
DreamBank	GPT2	31.9	1.4	137	-
WikiText2	GPT2	27.4	1.7	137	29.41
DreamBank	GPT2-Medium	26.7	1.4	380	-
WikiText2	GPT2-Medium	20.0	1.7	380	22.76
DreamBank	GPT2-Large	24.2	1.4	812	-
WikiText2	GPT2-Large	17.2	1.7	812	19.93
DreamBank	GPT2-XL	22.9	1.4	1610	-
WikiText2	GPT2-XL	15.5	1.7	1610	18.34
DreamBank	OLMo-1B	19.9	1.4	1180	-
WikiText2	OLMo-1B	12.4	1.7	1180	-
DreamBank	OLMo-7B	16.5	1.4	6890	-
WikiText2	OLMo-7B	8.7	1.7	6890	-

Analysis of the relation between length (number of tokens) and perplexity scores produced by GPT2 when considering DreamBank and Wikipedia data as a whole.

**Figure 3:**
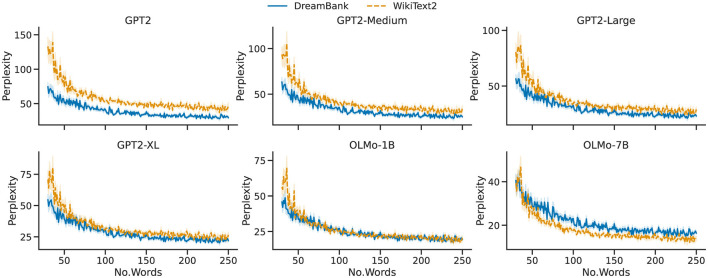
Perplexities by number of words per single item. Visualization of the interaction (mean and standard error, described by the shades) between the number of words (x-axis) and the perplexity scores (y-axis) produced by GPT2 for single dream reports and WikiText2 articles.

These results suggest that, considered as a whole corpus (i.e., a subsequent and unique string of text), Wikipedia is slightly easier to predict for all selected models. However, the main focus of our work is to understand if *single* dream reports are harder to model—i.e., are less *predictable*—than *single* Wikipedia articles, as these would generally be the input to any given LLM. Figure 3 offers a rather intuitive and straightforward answer to this question, by plotting the average perplexity score produced by each model (Y axis), given an instance with a defined number of words (X axis). In each diagram, the continuous blue line represents dream reports from DreamBank, while the dashed orange line represents articles from Wikipedia. Our analysis reveals that for all GPT2 models, the two two-dimensional distributions are significantly different from one another (*p* < 0.0001), and a random permutation test further showed that the one-dimensional distribution of the perplexity scores alone is too (*p* < 0.01). As the figure intuitively suggests, our analysis also conforms to the fact that, while always significant, the differences tend to fade as the model size increases. Looking at the Peacock test (Peacock, 1983; Fasano and Franceschini, 1987), we see how the score of GPT2, *D*=.34, slowly reduces passing from GPT2-Medium, *D* = 0.23, GPT2-Large, *D* = 0.19, and reaching *D*=.15 for GPT2-XL.

For the OLMo models, we observe a rather different trend. The two lines appear to overlap under OLMo-1B, and the GPT2 tendency seems inverted for OLMo-7B, with DreamBank's scores surpassing Wikipedia ones. This interpretation is confirmed by the statistical analysis. Under both models, we found a significant overall difference with the Peacock test (*p* < 0.0001). However, the random permutation analysis showed that the difference in perplexity scores is not significant for OLMo-1B. Moreover, the distance between the two distributions in OLMo-7B is notably small (*D* = 0.17), a rather surprising result considering that both OLMo have been exposed to (part of) Wikipedia during their training phase.

In summary, our results show that a large proportion of the LLMs under investigation found dream reports to be significantly more predictable than a more formal and structured text, such as Wikipedia articles. Most importantly, these models, namely the GPT2 ones, did not include any Wikipedia article in their training data, and hence have no advantages over dream reports. In the case of the two OLMo models, that did include some Wikipedia articles in their training data, and should hence have a clear advantage over dream reports, only the 7B version is overall better at modeling Wikipedia articles over dream reports.

### 3.2 Group analysis

The previous section provides consistent evidence that dream reports might be easier to model than more “standardised” strings of texts, such as Wikipedia articles. In this section, we study whether three directly measurable macro factors previously studied in the relevant literature also impact how well an LLM can model dream reports. The analysis takes into consideration five factors. The number of words per report (No. Words), year of collection, and three variables that were previously observed in the literature to produce qualitative changes in the content and structure of dream reports, namely gender, vision impairment, and clinical patients (Hall and Van De Castle, 1966; Schrdel and Reinhard, 2008; Wong et al., 2016; Kirtley, 1975; Hurovitz et al., 1999; Meaidi et al., 2014; Mota et al., 2014; Zheng et al., 2024). Lastly, this section focuses solely on GPT2 and OLMo-7B. This choice is motivated by the fact that they represent the models with the most marked preference for one of the two datasets.

**Figure 4:**
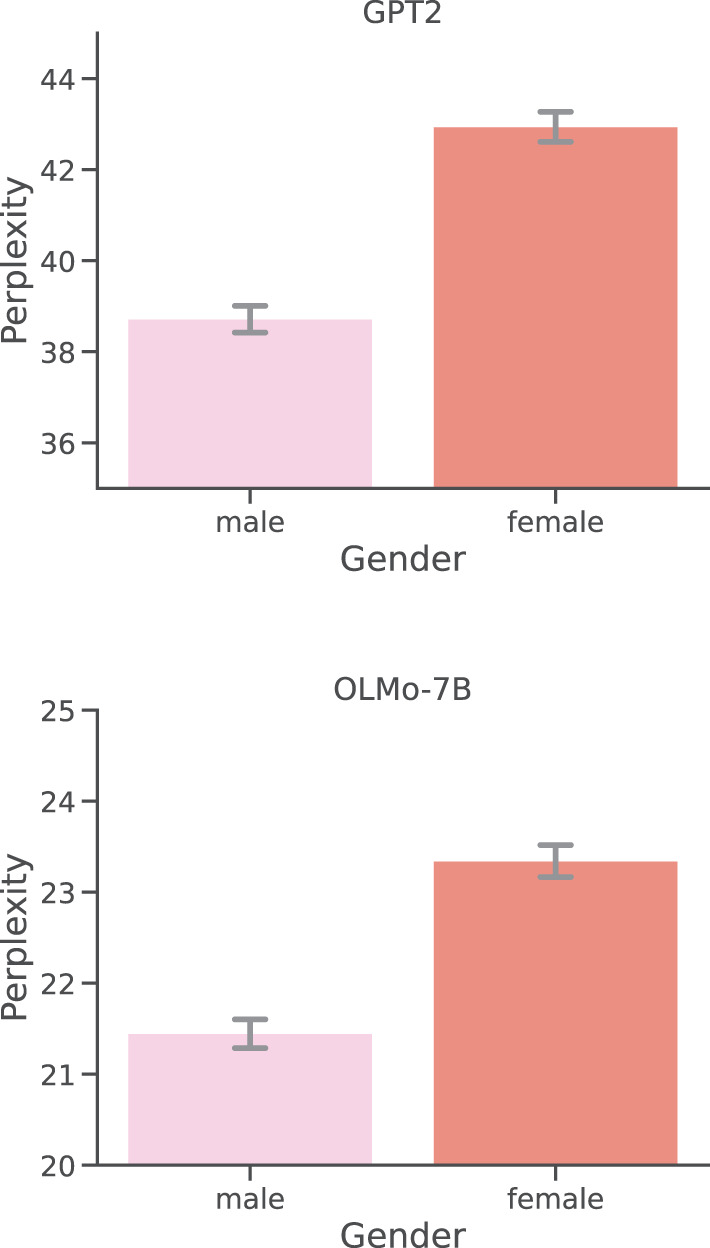
Per-report perplexities: gender. Distributions of perplexity scores obtained by GPT2 and OLMo-7B on DreamBank single dream reports grouped by the gender of the participants.

**Figure 5:**
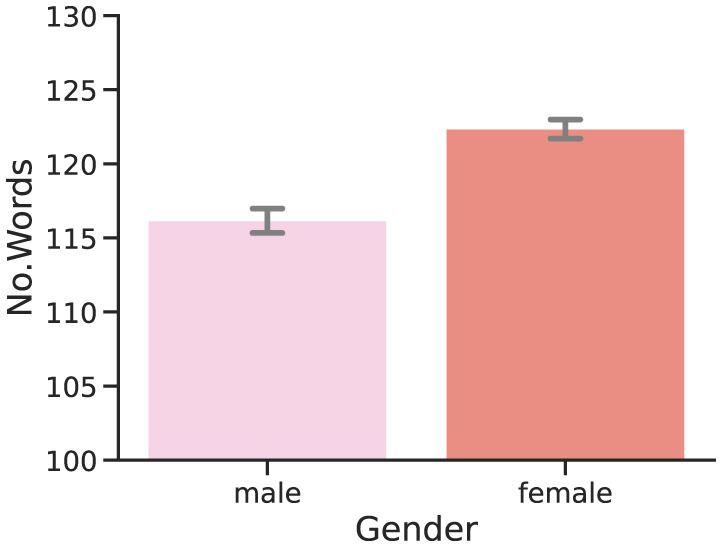
Per-report number of words: gender. Distributions of the word count of DreamBank single dream reports divided by the gender of the participants.

As hinted by Figure 3, our analysis has found a negative correlation between the number of words per report, and perplexity scores, both for GPT2 (ρ = - 0.33) and OLMo-7B (ρ = - 0.38), and both strongly significant (*p* < 0.0001). Observing lower perplexity scores for larger documents is not unexpected, since predicting a given word becomes easier as the context to guess said word becomes more abundant. While rather expected and explainable, this (co)relationship is likely more complicated than expected, as suggested by the relation between perplexity and word count in dream reports from participants of different genders. As shown in Figure 4, the perplexity scores produced by participants who identify themselves as male are significantly (both *p* < 0.01) lower, and are hence easier to model and predict for both GPT2 and OLMo-7B. However, as clearly shown in Figure 5, these reports are also significantly (*p* < 0.01) shorter than the ones produced by participants who identify themself as female, as already observed in other work (e.g., Mathes and Schredl, 2013). In other words, while from a general stance, shorter reports appear to entail higher perplexity, the trend seems to invert when taking into account the gender subgroup.

The effect of the year of data collection on the perplexity scores is also assessed with a correlation analysis. To this end, we converted the categorical framing of some instance (e.g., “1980s–1990s”), by simply finding the average a time-span (e.g., 1985). Instances with non-available dates were excluded from the analysis. The obtained dates, together with the original ones, are presented in Table 2. The result of the analysis suggests a negative correlation between the year of collection and the perplexity scores. In other words, as one might expect, reports produced in more recent years appear easier to model for GPT2, and hence tend to produce lower perplexity scores. However, while strongly significant (*p* < 0.0001), the effect was very weak for both GPT2 (ρ = -0.13) and OLMo-7B (ρ = -0.16).

**Table 2:** Conversion table for DreamBank's year of collection variable.

**DreamBank**	**Integer conversion**
1897–1918	1907
1912–1965	1938
1939	1939
1940–1998	1969
1940s–1950s	1945
1940s–1950s & 1990s	1960
1946–1950	1948
1948–1949	1948
1949–1964	1956
1949–1997	1973
1957–1959	1958
1960–1997	1978
1960–1999	1979
1962	1962
1963–1965	1964
1963–1967	1965
1964	1964
1968	1968
1970	1970
1970–2008	1989
1971	1971
1980–2002	1991
1985–1997	1991
1990–1999	1994
1990s	1990
1991–1993	1992
1992–1998	1995
1992–1999	1995
1995	1995
1996	1996
1996–1997	1996
1996–1998	1997
1997	1997
1997–1999	1998
1997–2000	1998
1997–2001	1999
1998	1998
1998–2000	1999
1999	2010
1999–2000	1999
1999–2001	2000
2000	2000
2000–2001	2000
2001–2003	2002
2003–2004	2003
2003–2005	2004
2003–2006	2004
2004	2004
2007–2010	2008
2009	2009
2010–2011	2010
?	NaN
Late 1990s	1998
Mid-1980s	1985
Mid-1990s	1995

**Figure 6:**
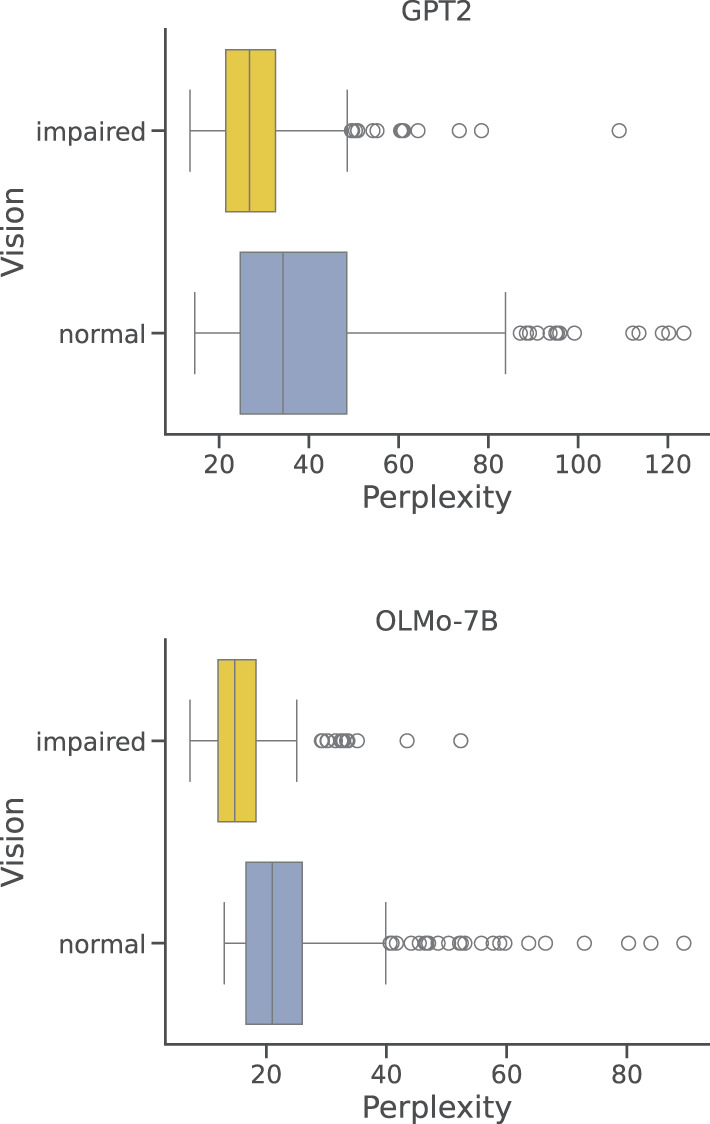
Per-report perplexities: vision impairment. Distributions of perplexity scores obtained by GPT2 and OLM0-7B on DreamBank single dream reports grouped by vision impairment of the participants.

Among DreamBank's series, there are two that collect reports from several blind participants, both males and females, for a total of 285 dream reports. To compare this restricted set of reports with the one produced by normally-sighted individuals, we have sampled a set of reports from DreamBank that has the same range of perplexity scores observed for blind participants. Just like for the general and gender-based results, these two sets show to be significantly different (*p* < 0.0001) when considered as two-dimensional distributions (as in Figure 3); however, when taken separately, only the perplexity score turned out to be significantly different (*p* < 0.01). Figure 6 summarizes the differences in the perplexity scores distributions obtained for reports produced by visually impaired and normally sighted participants. As shown, even when sampling from a limited range of items, perplexity scores for visually impaired participants are on average considerably lower and have a remarkably smaller variance, especially when encoded with GPT2.

**Figure 7:**
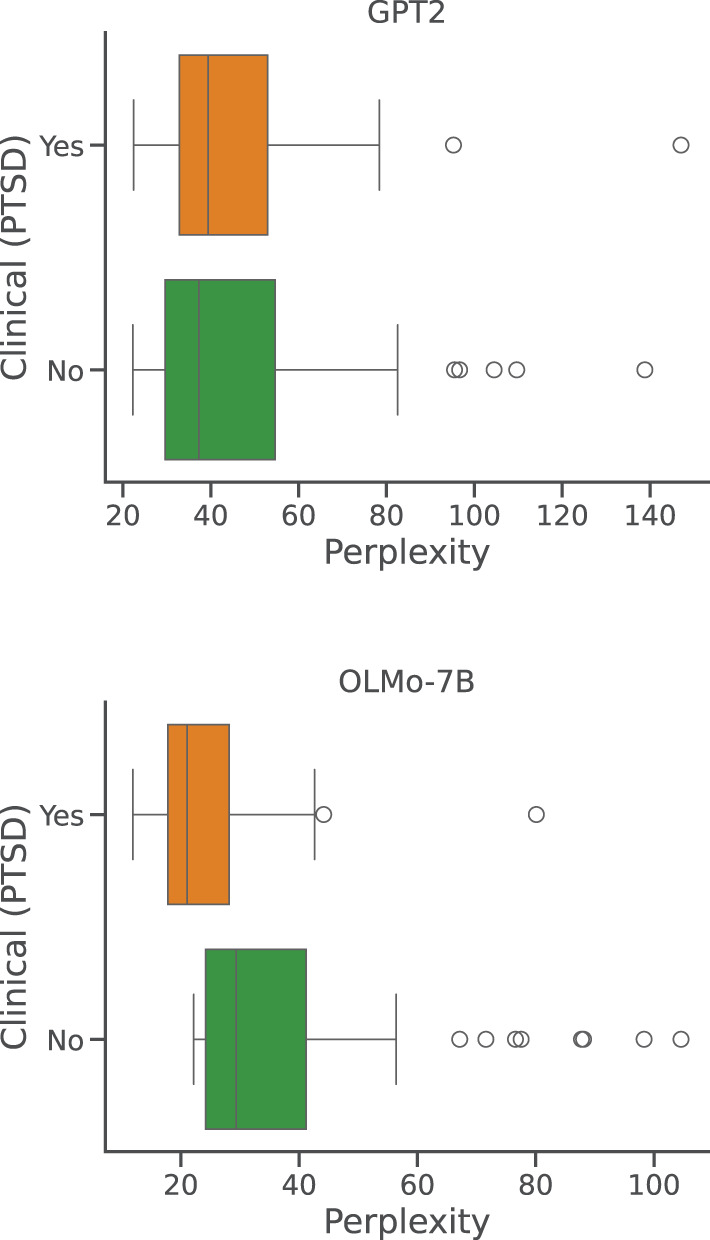
Per-report perplexities: clinical patient. Distributions of perplexity scores obtained by GPT2 and OLMo-7B on DreamBank single dream reports grouped by clinical condition.

Lastly, we consider a very small set (circa 70 instances) of reports belonging to a subject diagnosed with post-traumatic stress disorder (PTSD), a veteran of the Vietnam War. We follow the same sampling procedure and overall analysis described in the previous paragraph for the visually impaired participants, summarized in Figure 7. As suggested by the two diagrams, the difference in perplexity scores is significant only under the OLMo-7B model (*p* < .01). Indeed, these results are limited by the small size of sample, and are hence harder to frame and contextualize. We note that the results from GPT2 appear in line with the last experiment in Bertolini et al. (2024a), where the authors showed that a small LLM trained to classy dream for emotional content, using a report from healthy participants, performed well on this same set, despite being out of distribution. In contrast, the results from OLMo-7B appear in line with the work suggesting that clinical participants produce dream reports that significantly differ from healthy participants, as suggested by Mota et al. (2014). It is interesting to note that the work from Mota et al. (2014) largely relies on graphs-based analysis and patterns, and that transformers (Vaswani et al., 2017), the neural network at the base of most LLMs, can be considered as a special case of graph neural networks (Veličković, 2023). It is possible that a large enough model could locally and implicitly represent the same type of graph that is useful to distinguish between clinical and healthy participants.

## 4 Discussion

A growing amount of work has adopted NLP tools to investigate and annotate dream reports (see Elce et al., 2021; Bertolini et al., 2024a; Cortal, 2024 for more details). Many of these approaches rely on neural models of various dimensions, trained on large text corpora scraped from the web (Radford et al., 2019). Since a consistent body of evidence has shown that the structure and semantic content of dream reports can significantly differ from other types of textual transcripts (see Altszyler et al., 2017; Bulkeley and Graves, 2018; Zheng and Schweickert, 2023, inter alia), it is important to understand, and possibly quantify, if and how much these differences impact the ability of NLP tools to model and interpret rather specific strings of text, such as dream reports. This is especially relevant and important when adopting off-the-shelf and unsupervised models and methods, as already hinted by Bertolini et al. (2024a).

In this work, we adopted a set of large language models (LLMs) from the GPT2 and OLMo family, to investigate these issues. More specifically, we studied how well they can model and predict dream reports, compared to a more “standard” text, like Wikipedia articles, using perplexity as a measure of uncertainty. Our results have shown how most LLMs produce significantly lower perplexity scores (hence better) for single dream reports than for single Wikipedia articles. The only exceptions to this trend were observed in the two OLMo models. However, these models contained part of Wikipedia in their training data, and hence had a notable advantage. Moreover, we found only a partial significance in the smaller model (OLMo-1B), and a marginal advantage for the larger model (OLMo-7B).

These findings paint a clear picture. A picture where LLMs, or at least the ones tested in this work, do not seem to struggle at all with processing dream reports, nor do they seem more “surprised” by dream reports than they are by Wikipedia articles. In the literature, a consistent research line tends to associate dreams and their reports with bizarreness (Rosen, 2018), entailing a significant deviation from normal experience, whatever that might be. This view appears in clear contrast with our findings, as they indicate that for LLMs, dream reports are as “predictable” as the “the norm”, at least in the form of Wikipedia text. This might come as unexpected but it is likely due to what is *our* compass for dream reports bizarreness: reality. Aside very specific pathological cases and scenarios, we are generally capable of distinguishing a bizarre or absurd event from reality. LLMs, on the other hand, are machines designed and trained to encode or generate text, regardless of its truthiness or correctness, and can in fact frequently struggle even with identifying simple and established facts (Wang et al., 2024). This does not mean that LLMs can not or should not be used in dream research. On the contrary, our work suggests they *can* handle these unique strings of text. However, these results show that when using LLMs in this line of research, we should be extremely careful in projecting *our* definition and understanding of the mind and world onto these tools. One of these definitions might in fact be bizarreness, for which humans and LLMs might have a very different “concept”. Future work will have to focus on providing more insight into the existing relation between perplexity, or other mathematically measurable metrics, to carefully operationalised human concepts such as bizarreness, surprisal, or predicability.

Indeed, the main findings of this work suggest that dream reports are not a unique and unpredictable class of textual strings per se. However, just like Wikipedia's articles, some *can be* harder to model and predict. The second part of the work has hence proposed a set of analyses to understand which features of a report—or their author—might have an impact on the model of choice. The focus was on two specific models, namley GPT2 and OLMo-7B, and five variables immediately measurable from the adopted dataset: word count (No.Words), gender, year of collection, visual impairments, and mental health. We focused our attention on these models since they produced the most marked preference for dream reports or Wikipedia, while the choice of variables was based on group differences previously found in the literature.

The correlation analysis found a (rather expected) negative interaction between the number of tokens contained in a report (No.Words), and the perplexity scores produced by each model, which was also found for Wikipedia articles. However, further analyses suggested that the observed effect might be largely influenced by a consistent set of outliers, with very low perplexity scores. In other words, there appears to be another mediating variable influencing how challenging is for an LLM to model a dream report. Overall, the analysis further weakened the hypothesis that dream reports are rather unique strings of texts. All DreamBank's results, from the negative correlation to the outliers' effect and the shape of the distribution, found a strong match in the results produced by the models when tested on Wikipedia data.

The results based on gender and visual impairment further challenged the strength of the negative correlation between perplexity and word count. On the one hand, under both models, reports from blind participants did resulted in significantly lower perplexity, but no significant effect was found between the two groups in terms of reports' length. Even more strikingly, in the case of gender, the group with significantly lower perplexity scores (i.e., male) turned out to produce also significantly shorter reports. Again, these results patterns were stable across the two models. These discrepancies suggest that what really has an impact on the ability of the model to process a given report might have less to do with the number of words and more with the *type* of words in a report. A similar conclusion was also proposed in Bertolini et al. (2024a). Using an out-of-distribution ablation experiment, it was shown that leaving a specific DreamBank series out of training made it difficult for the model to handle a specific emotion (e.g., “happiness” for the Bea 1 series.). The authors noted that this could not be simply explained by the number of instances in the training data, and was likely related to the specific vocabulary used in that specific series to describe that particular emotion.

The work also adds more evidence to the existing body of scientific knowledge showing how the gender of a participant might impact the related dream report (Hall and Van De Castle, 1966; Schrdel and Reinhard, 2008; Wong et al., 2016; Zheng et al., 2024). While repeatedly observed, these differences were mainly constrained to a report's semantic content and/or grammatical structure, such as a reference to a specific emotion, use of violent language, or part-of-speech use. This work suggests that the observed distinction might have a very tangible effect since reports produced by male dreamers were found to be significantly easier, on average, to model by both GPT2 and OLMo. This likely suggests that the distinction is even deeper than previously noted, and might include a combination of content, vocabulary, and structure.

A possible explanation for the observed gender-based difference might come from the data used to train these models, which is largely scraped from the internet. Multiple reports and preliminary studies have identified a worldwide disproportion in internet usage that disadvantages female users (Breen et al., 2025). This disproportion might not be limited to internet usage. For instance, in 2012, a Wikipedia blogpost estimated that up to 90% of its editors were men^4^, a number later confirmed by a survey in 2018^5^. More recently, researchers have used corpus-linguistics and word embedding to show that within (a large English-based corpus extracted from) the internet, the concepts of “people” and “person” do not appear to be gender neutral, but are more aligned with the concept of “men” (Bailey et al., 2022). This misalignment was also observed in machine-human interaction. A preliminary work found that ChatGPT was more frequently perceived as male rather than female on a variety of tasks (Wong and Kim, 2023). In other words, it is possible that LLMs might find male-generated dream reports easier to model and predict because they have been primarily trained on male-generated data.

Results suggesting that blind dreamers produced more predictable reports seem more difficult to frame in the current literature and knowledge. Multiple pieces of evidence across time have shown how blind participants express a significantly lower amount of visual features in their reports, predominantly presenting auditory, tactile and olfactory reference (Kirtley, 1975; Hurovitz et al., 1999; Meaidi et al., 2014; Zheng et al., 2024). However, Meaidi et al. (2014) showed that these differences can significantly vary between congenitally and late blind participants, and both series contain a mixture of congenitally and non-congenitally blind participants (although most have been for more than 20 years). A possibility might be that maintaining access to the visual modality while dreaming allows for a larger degree of abstraction and variance of dream content, leading sighted participants to generate more diverse reports, that can result in harder sequences to predict for the model. Regardless of this hypothesis, it is important to notice that, since the two series contain reports produced by several individuals—approximately thirty—with an age window spanning from 24 to 70, and remarkably different backgrounds, it is unlikely that a single participant drives the observed difference in perplexity scores.

Concerning the year of collection of each report, one might find the observed small effect as unexpected, considering that many reports were collected at a time when the internet existed only in the minds of visionary scientists and writers. However, this might be explained by the fact that the internet is a collection of extremely heterogeneous documents, that obviously include very old textual instances. It is hence possible that, while specific reports did not leak into the training data, their vocabulary and style might very well have. In other words, the model might have also been exposed to the form and vocabulary used in older reports.

Overall, we believe that this work adds an important piece of evidence to the literature investigating differences in dream experiences from different groups. We have long been aware that reports produced by participants with different gender or visual impairment tend to present significantly different content—and hence different word distributions. The experiments proposed in this work, however, further suggest that these differences are not limited to *which* words these groups use, but also *how* these groups use words, and that these differences in word usage as a measurable impact on current NLP tools.

To conclude, it is important to notice that this work has three main limitations. First of all, while OLMo training set is fully open-source, WebText, the dataset used to train GPT2, is not, and it is hence harder to estimate possible data leakage from DreamBank. That is, whether a part of the test data used in this work was also included in the training data for the model. In their work, Radford et al. (2019) note that training text for GPT2 was scraped following outbound links from Reddit, with at least 3 karma, and one link connecting Reddit to DreamBank. However, the link reached the main page of DreamBank, which does not allow scraping dream reports. As shown by example codes (e.g., here^6^), the main solution to acquire dream reports from DreamBank is to iteratively sample them via the random sample page, which requires actively entering specific settings—such as series or number of words—to print out a set of reports. In other words, it seems quite unlikely that a consistent part of the test data for this work was in fact also included in the training data for GPT2. Future work will have to focus on models like OLMO, where the full extent of the training data is available. This would ensure better comparison and understanding of other relevant phenomena, such as whether the difference in perlocutionary scores might be connected to a specific type of documents, like Wikipedia articles of web-scraped dialogue, and with what strength. Second, the language of tested items was limited to English. Third, the adopted dream report dataset, DreamBank, is not fully transparent about the extent to which the reports were manipulated. The extended amount of grammatical errors and informal structures/forms found upon a manual inspection of a (limited) set of reports suggested that the data went through a very limited manipulation, but this can not be widely confirmed. Future work will have to investigate how strongly these findings can be generalized to other languages and dream datasets, as well as to provide a more detailed explanation of what might make a report more complex to predict for a current LLMs, taking more into consideration semantic content and syntactic structures.

## 5 Conclusion

This study has provided compelling evidence that dream reports are not the unpredictable textual entities they were once thought to be. By employing a set of large language models to analyze and predict the textual content of dream reports and compare it with standardized texts from Wikipedia, the research has shown that dream reports are, on average, more predictable than Wikipedia articles. This finding challenges the assumption that dream content is too peculiar or bizarre for models trained on web-based corpora. Additionally, the study has uncovered intriguing differences in predictability related to the gender and visual impairment of dream report authors, suggesting that these factors significantly influence the language models' performance. These results not only contribute to our understanding of dream report characteristics but also have implications for the use of natural language processing tools in dream research. The insights of the presented study into the predictability of dream reports and the factors that affect it open the path for future research into the complex ways in which different groups express their dream experiences.

## Data Availability

The original contributions presented in the study are included in the article/supplementary material, further inquiries can be directed to the corresponding author.

## Footnotes

^1^ https://huggingface.co/docs/transformers/perplexity

^2^ https://dreambank.net/

^3^We use the wikitext2-v1-raw subset from https://huggingface.co/datasets/Salesforce/wikitext.

^4^ https://diff.wikimedia.org/2012/04/27/nine-out-of-ten-wikipedians-continue-to-be-men/

^5^ https://meta.wikimedia.org/wiki/Community_Insights/2018_Report/Contributors

^6^ https://github.com/mattbierner/DreamScrape
